# Exploration of New-Generation Herbicide Combination for Direct-Seeded Rice Cultivation in India

**DOI:** 10.3390/plants15213486

**Published:** 2026-11-08

**Authors:** Arijit Garai, Biswajit Pramanick, Dhirendra K. Roy, Santosh Kumar Singh, Nilanjaya, Vipin Kumar, Md. Abbas Ahmad, R. K. Ranjan

**Affiliations:** 1 Department of Agronomy, Dr. Rajendra Prasad Central Agricultural University, Pusa 848125, Bihar, India; 2 Department of Soil Science, Pandit Deendayal Upadhyay College of Horticulture & Forestry, Piprakothi, Dr. Rajendra Prasad Central Agricultural University, Pusa 848125, Bihar, India; 3 Department of Soil Science, Dr. Rajendra Prasad Central Agricultural University, Pusa 848125, Bihar, India; 4 Department of Genetics and Plant Breeding, Dr. Rajendra Prasad Central Agricultural University, Pusa 848125, Bihar, India; 5 Department of Entomology, Dr. Rajendra Prasad Central Agricultural University, Pusa 848125, Bihar, India; 6 Department of Plant Pathology and Nematology, Dr. Rajendra Prasad Central Agricultural University, Pusa 848125, Bihar, India

**Keywords:** dry direct-seeded rice, florpyrauxifen-benzyl + penoxsulam, triafamone, weed control efficiency, yield

## Abstract

Weed management has emerged as the main constraint to the successful adoption of climate-resilient dry direct-seeded rice (DDSR) in India. Entire-crop failure has been reported in many direct-seeded rice cases, which is the main constraint to adopting this climate-resilient technology among Indian farmers. A two-year field experiment was conducted during the rainy seasons of 2023 and 2024 to identify effective herbicide combinations for the control of diverse weed flora in DDSR. The conventionally recommended herbicides such as bispyribac-sodium, pyrazosulfuron-ethyl, etc., and recently introduced herbicides like florpyrauxifen-benzyl, triafamone, etc., which have not been explored extensively in the DDSR production system in India, were evaluated. It was found that weed management practices considerably influenced the weed density, weed biomass, weed control efficiency, and grain yield in DDSR. Among the herbicide combinations evaluated, pendimethalin at 1000 g/ha as pre-emergence followed by florpyrauxifen-benzyl (1.31% *w*/*w*) at 15.3 g/ha + penoxsulam (2.1% *w*/*w*) at 20 g/ha as post-emergence at 25 days after sowing provided the most effective broad-spectrum weed control by recording the lowest overall weed density and biomass throughout the crop growth period. Whereas pendimethalin followed by bispyribac-sodium + pyrazosulfuron-ethyl was the most effective against *Cyperus* spp., pendimethalin followed by triafamone + ethoxysulfuron recorded the lowest density and biomass of *Cynodon dactylon*. Treatment T_4_ recorded weed control efficiencies of 88.0, 88.6, and 85.0% at 30, 60, and 90 days after sowing, respectively, and produced a grain yield of 4.67 Mg/ha, which was statistically at par with the weed-free treatment (4.80 Mg/ha). The sequential application of pendimethalin followed by florpyrauxifen-benzyl + penoxsulam provided effective broad-spectrum weed control and maintained rice productivity comparable to the weed-free treatment. Therefore, this herbicide combination is recommended for weed management in dry direct-seeded rice (DDSR) in India.

## 1. Introduction

Rice is a significant component of India’s food production system, and keeping up its productivity under mounting climatic and resource constraints has become a crucial agricultural priority [1]. In this context, dry direct-seeded rice (DDSR) is emerging as a climate-resilient production approach providing an alternative to conventional puddled transplanted rice [2,3]. However, the successful adoption of DDSR is challenged by a number of agronomic challenges, and weed management is one of the critical ones [4,5]. Consequently, weeds can become highly competitive with the crop from the early stages of growth, resulting in substantial reductions in crop growth and productivity. Under severe infestation, weed competition may even lead to complete crop failure [6]. The diverse and dynamic weed flora associated with DDSR further complicates weed management. Grassy weeds such as *Echinochloa crusgalli* (L.) *P. Beauv.*, *Cynodon dactylon* (L.) Pers., *Digitaria sanguinalis* (L.) Scop., etc., sedges including *Cyperus rotundus* L., *C. difformis* L., etc., and broad-leaved weeds such as *Eclipta alba* (L.) Hassk., *Alternanthera sessilis* (L.) R.Br. ex DC., *Phyllanthus niruri* L., etc., may occur simultaneously in the crop, particularly in the eastern Indian rice bowl area [2,4]. An effective weed management program for DDSR must involve timely and broad-spectrum suppression of a diversity of weed groups over the critical period of crop–weed competition.

Herbicides have become indispensable for weed management in dry direct-seeded rice (DDSR), enabling timely and effective weed control over large areas [7]. In dry direct-seeded rice (DDSR), weeds emerge alongside the crop and may continue emerging throughout the growing season, resulting in diverse weed flora with varying growth habits and herbicide sensitivities. Consequently, a single herbicide application may not provide adequate season-long weed suppression. Although conventional herbicides such as bispyribac-sodium and pyrazosulfuron-ethyl are widely used for weed management in rice [3], changing weed spectra necessitate the evaluation of alternative herbicide combinations and sequential application strategies. Conventional herbicides such as bispyribac-sodium and pyrazosulfuron-ethyl are widely used for weed management in rice [3]; however, the changing weed spectrum and the need for broader and more consistent control necessitate the evaluation of alternative herbicide combinations and sequential application strategies [8]. Accordingly, the herbicide sequences in the present study were selected to provide complementary weed-spectrum coverage through sequential pre- and post-emergence control. Pendimethalin was applied pre-emergence to suppress early emerging grasses and broad-leaved weeds, while the subsequent post-emergence herbicides were selected for their complementary activity against escaped and later-emerging weeds. Bispyribac-sodium + pyrazosulfuron-ethyl, penoxsulam, triafamone + ethoxysulfuron, and florpyrauxifen-benzyl + penoxsulam differ in their modes of action and weed control spectra, providing opportunities for broader and more sustained weed suppression. Newer herbicides such as florpyrauxifen-benzyl and triafamone offer additional options for managing complex weed flora [9]. Their application at appropriate crop and weed growth stages was therefore intended to control successive weed flushes, reduce crop–weed competition, and maintain effective weed suppression throughout the critical period of rice growth.

However, the comparative performance of these herbicides, particularly their sequential pre-emergence (PRE) followed by post-emergence (POST) programs, under dry direct-seeded rice (DDSR) conditions in India remains insufficiently documented. Most previous studies have evaluated individual herbicides or conventional herbicide programs, while limited information is available on the comparative efficacy of newer herbicide combinations integrated into sequential programs for managing the diverse and temporally variable weed flora of DDSR. In particular, the relative effectiveness of a PRE application of pendimethalin followed by different POST herbicide options, including the recently introduced florpyrauxifen-benzyl + penoxsulam combination, has not been adequately compared under the same field conditions. Since weed emergence occurs in successive flushes in DDSR, the timing and sequence of herbicide application are critical for achieving broad-spectrum and season-long weed suppression. Pendimethalin applied PRE can suppress the initial flush of weeds, whereas an appropriately timed POST application can control escaped and later-emerging weeds [10]. Therefore, comparing different PRE–POST herbicide sequences under identical field conditions can help identify programs that provide more consistent weed suppression, reduce weed biomass, and minimize yield losses.

Thus, the present study was undertaken to comparatively evaluate conventional and newly introduced sequential herbicide programs, with particular emphasis on the PRE pendimethalin followed by different POST herbicide combinations, for managing diverse weed flora in DDSR under Indian conditions. A two-year field experiment was conducted during the rainy (*kharif*) seasons of 2023 and 2024 to quantify treatment effects on weed density, weed biomass, weed control efficiency, weed index, and rice grain yield. Primary importance was placed on identifying herbicide sequences that would provide broad-spectrum and season-long weed control, and that would also provide grain yields comparable to those obtained under weed-free conditions. The study also aimed to determine treatment-specific responses against important grassy, sedge, and broad-leaved weed species and to develop an effective herbicide-based strategy for higher productivity of dry direct-seeded rice.

## 2. Results

### 2.1. Weed Density

The weed flora was dominated by grasses, sedges and broad-leaved weeds, with *Echinochloa crusgalli*, *Cynodon dactylon*, and *Digitaria sanguinalis* as the grasses; *Cyperus rotundus* and *C. difformis* as the sedges; and *Eclipta alba*, *Alternanthera sessilis* and *Phyllanthus niruri* as the broad-leaved weeds. The data on weed flora density for 2023 and 2024 were pooled across the two years and are presented in Figure 1. The density of the weed flora varied greatly with different treatments for all of the weed species at 30 days after sowing (DAS) (Figure 1). Whereas the lowest weed density was recorded in the weed-free treatment (T_6_) by completely suppressing the emergence of all of the tested weed species, the highest weed density was recorded in the unweeded control (T_7_). In most of the herbicidal treatments, T_4_ (pendimethalin 1000 g/ha as pre-emergence followed by florpyrauxifen-benzyl 1.31% *w*/*w* 15.3 g/ha + penoxsulam 2.1% *w*/*w* 20 g/ha at 25 DAS) was the most efficient treatment by suppressing the density of *E. crusgalli*, *C. dactylon*, *D. sanguinalis*, *C. rotundus*, *C. difformis*, *E. alba*, *A. sessilis* and *P. niruri* to the level of 1.17, 1.81, 1.86, 2.02, 2.08, 1.17, 1.22 and 0.71, respectively, followed by T_1_ (pendimethalin 1000 g/ha as pre-emergence followed by bispyribac-sodium 25 g/ha + pyrazosulfuron-ethyl 15 g/ha at 25 DAS) and T_5_ (pendimethalin 1000 g/ha as pre-emergence followed by penoxsulam 20 g/ha at 12 DAS + bispyribac-sodium 25 g/ha at 25 DAS). However, T_2_ (pendimethalin 1000 g/ha as pre-emergence followed by penoxsulam 20 g/ha at 25 DAS) proved to be the least efficient by allowing higher weed density, especially *C. rotundus* (3.52) and *C. difformis* (3.80). Thus, the results showed that the pre- and post-emergence herbicides, particularly T_4_, significantly reduced the density of broad-leaved weeds, sedges, and grasses as compared to the weedy control (T_7_) at 30 DAS.

A similar trend was observed at 60 DAS (Figure 2). At this stage also, T_4_ resulted in the best weed control among the chemical weed control treatments by suppressing the densities of *E. crusgalli* (2.33), *D. sanguinalis* (5.0), *E. alba* (2.0), *A. sessilis* (1.33), and *P. niruri* (0.67). Similarly, T_1_ also showed to be effective by reducing the densities of *C. rotundus* (2.0), *C. difformis* (4.0), and *P. niruri* (1.0). On the other hand, T_2_ resulted in higher densities of *C. rotundus* (15.0) and *C. difformis* (14.0), depicting its lower efficacy against sedges. Even at the milking stage of rice, i.e., 90 DAS, the lowest weed density was recorded in T_4_ with respect to *E. crusgalli* (4.33), *D. sanguinalis* (7.33), *E. alba* (2.33), *A. sessilis* (2.33), and *P. niruri* (1.0) (Figure 3). Moreover, T_1_ resulted in effective weed control against *C. rotundus* (3.67) and *C. difformis* (5.33). However, T_2_ recorded higher densities of *C. rotundus* (17.0) and *C. difformis* (16.67). Thus, it can be stated that T_4_ depicted the most efficient treatment among the chemical weed management treatments, which was found to be on par with the weed-free treatment (T_6_).

### 2.2. Weed Dry Weight

Weed dry weight (g/m^2^) increased progressively with advancement of crop growth under all of the chemical weed management treatments, viz., the higher the crop growth stage, the higher was the infestation; however, the extent of occurrence varied with respect to treatments and weed category (Table 1). The lowest dry matter of grasses (2.30, 2.73 and 3.15 g/m^2^), sedges (2.49, 3.27 and 3.63 g/m^2^) and broad-leaved weeds (1.67, 1.94 and 2.06 g/m^2^) was registered at 30, 60 and 90 DAS, respectively, under T_4_ and the treatment was found to be the most effective in suppressing the infestation of broad-leaved weeds as compared to other herbicidal treatments. Under T_1_, the dry matter of grasses and sedges was recorded as 3.99 and 3.13 g/m^2^, respectively, at 90 DAS, which was found to be the second highest among the treatments. The herbicidal treatments T_3_ and T_5_ showed moderate weed control, whereas T_2_ recorded the highest weed dry weight among the chemical treatments, especially for grasses and sedges at later growth stages. Though the chemical treatment T_4_ showed better and broad-spectrum weed suppression, its performance was found to be lower than that of the weed-free treatment, T_6_. Thus, it may be stated that none of the chemical treatments was found to be as effective as the complete weed-free treatment, T_6_. The progressive increases in weed density under the chemical treatments suggested that the residual effects of the herbicides were not sufficient to maintain the suppression of weeds until 90 DAS; however, T_4_ showed the best performance, particularly in terms of grasses, sedges and broad-leaved weeds.

### 2.3. Weed Control Indices

The weed control efficiency (WCE%) was found to be greatly influenced by the weed management practices and decreased to a certain extent with the advancement of crop growth (Table 2). The data were pooled over two years for presentation in this article and showed that among the chemical treatments, the WCE of T_4_ was found to be higher than the other treatments, registering 88.0, 88.6 and 85.0% at 30, 60 and 90 DAS, respectively. Similarly, the second-highest WCE was recorded under T_1_, ranging from 84.7 to 85.6% up to 60 DAS; however, it decreased to 79.6% at 90 DAS. The WCE under T_5_ ranged from 79.1 to 73.4% for the entire growth period, registering relatively higher efficacy at 60 DAS (81.8%), whereas a moderate level of control was registered under T_3_. On the other hand, the lowest WCE was recorded under T_2_, which ranged from 68.3 to 59.0%. As expected, the WCE under the complete weed-free treatment (T_6_) remained at 100% throughout the crop growth period. The lowest weed index (2.7%) was recorded under T_4_, followed by T_1_ (7.9%), T_5_ (13.5%), T_3_ (17.5%), and T_2_ (24.0%), with the highest value (46.0%) recorded under the unweeded control (Table 2). Thus, it can be stated that the combination of herbicides as in T_4_ registered the best chemical weed management option, which provided excellent weed control and hence minimized the yield loss as compared to other treatments.

### 2.4. Growth Parameters

Weed management treatments markedly influenced plant growth, tillering, and dry matter accumulation of rice as shown in the pooled data over two years of study (Figure 4). Among the chemical herbicide treatments, T_4_ (pendimethalin *fb* florpyrauxifen-benzyl + penoxsulam) recorded the highest values for all three growth parameters and was therefore the best-performing herbicide treatment. Plant height under T_4_ increased from 73.6 cm at 60 DAS to 92.4 cm at 90 DAS, compared with 53.9 and 69.0 cm, respectively, under the unweeded control (T_7_), representing increases of 36.5 and 33.9%. Similarly, T_4_ produced 282 and 244 tillers/m^2^ at 60 and 90 DAS, which were 46.9 and 52.5% higher than T_7_ (192 and 160 tillers/m^2^). Dry matter accumulation under T_4_ reached 468 and 811 g/m^2^ at 60 and 90 DAS, respectively, corresponding to increases of 70.2 and 90.8% over the unweeded control (275 and 425 g/m^2^). The weed-free treatment (T_6_) recorded the maximum crop growth overall, with 75.0 and 96.1 cm plant height, 286 and 250 tillers/m^2^, and 476 and 839 g/m^2^ dry matter at 60 and 90 DAS, respectively. However, T_4_ remained statistically comparable with T_6_ for most growth attributes, indicating that the pendimethalin *fb* florpyrauxifen-benzyl + penoxsulam sequence effectively minimized weed interference and maintained crop growth close to the weed-free condition. In contrast, continuous weed competition in T_7_ substantially restricted crop development, particularly dry matter accumulation during the later growth stage.

### 2.5. Yield Attributes and Yield

Weed management treatments substantially influenced the yield attributes of dry direct-seeded rice (Table 3). Panicle density and grains per panicle were significantly affected, whereas test weight remained statistically non-significant. Among the herbicide treatments, T_4_ (pendimethalin *fb* florpyrauxifen-benzyl + penoxsulam) recorded the highest panicle density (236 m^2^) and grains per panicle (136), closely followed by the weed-free treatment T_6_ (240 m^2^ and 144 grains per panicle, respectively). Compared with the unweeded control (T_7_), T_4_ increased panicle density and grains per panicle by 56.3 and 58.1%, respectively. T_4_ also recorded a test weight of 23.6 g, compared with 22.2 g under T_7_, although the treatment differences were non-significant. The lowest panicle density (151 m^2^) and grains per panicle (86) were recorded under T_7_, reflecting the strong adverse effect of uncontrolled weed competition on reproductive development. Among the chemical treatments, T_4_ was superior to T_1_–T_3_ and T_5_, with T_1_ recording 222 panicles per m^2^ and 131 grains per panicle, while T_2_ showed comparatively lower values (197 panicles per m^2^ and 121 grains per panicle). Overall, the greater panicle production and grain number under T_4_ indicate that effective weed suppression during crop establishment and reproductive development helped reduce weed-induced competition and improved the formation of productive sinks.

The grain and straw yields of the test treatments were found to be greatly influenced by the weed management practices (Table 4). The maximum grain yield (4.67 Mg/ha) was recorded under T_4_, which was 80.3% higher than the unweeded control. It was also found to be statistically at par with the complete weed-free treatment (T_6_; 4.80 Mg/ha). Notably, the grain yield under T_4_ was only 2.7% lower than that of T_6_, which indicated that the treatment performed exceedingly well not only in terms of WCE but also in terms of yield production. Considering the other herbicidal treatments, T_1_ (4.42 Mg/ha) recorded the second-highest grain yield, followed by T_5_ (4.15 Mg/ha), T_3_ (3.96 Mg/ha), and T_2_ (3.65 Mg/ha), and each of them recorded 70.7, 60.2, 52.9, and 40.9% higher yields than the unweeded control, respectively. It is also evident from the data that the grain yield advantage of T_4_ over T_2_ was as high as 27.9%. This indicated the importance of combining herbicides with different modes of action for the effective management of the diverse weed flora, particularly when compared to the use of a single post-emergence herbicide. Similar trends were observed for the straw yield, wherein the maximum straw yield (6.22 Mg/ha) was recorded under T_6_ followed by T_4_ (6.01 Mg/ha), whereas the lowest yield (3.85 Mg/ha) was recorded under the unweeded control. Overall, the harvest index ranged from 40.2 to 43.7%, with the highest value recorded under T_4_. Thus, it can be concluded that the combination of herbicides as in T_4_ was found to be the best among the chemical weed management options, as it not only provided excellent weed control but also produced grain yield almost comparable to the complete weed-free treatment while significantly outyielding the unweeded control.

### 2.6. Economic Analysis

The economic analysis of the weed management treatments indicated that T_4_ was the best option among the chemical weed management treatments (Table 5). Though T_4_ involved a slightly higher cost of cultivation as compared to T_1_–T_3_, it fetched the maximum gross returns (USD 1063/ha) and net returns (USD 657/ha) among the chemical treatments. In fact, it also recorded the highest B:C ratio (1.62), which indicated that the cost incurred on the herbicides was compensated by the additional returns. The second position in terms of net returns (USD 618/ha) and B:C ratio (1.57) was recorded by T_1_, followed by T_5_ and T_3_, whereas the lowest gross returns (USD 827/ha) and net returns (USD 443/ha) were recorded under T_2_. Significantly, T_4_ was also found to be more economical than the complete weed-free treatment, T_6_. Even though the latter recorded marginally higher gross returns (USD 1105/ha) than T_4_, its net returns (USD 572/ha) were lower due to higher cost of cultivation (USD 533/ha) and, consequently, a lower B:C ratio (1.07). Thus, it can be concluded that under the prevailing conditions, T_4_ was the most economical treatment among the chemical weed management options, as it not only registered higher net returns but also a higher B:C ratio as compared to T_6_. Overall, T_4_ performed the best in terms of weed control, yield, and economics and therefore can be recommended as the best chemical weed management option for rice cultivation.

## 3. Discussion

### 3.1. Weed Density

The weed community in the dry direct-seeded rice field was diverse, comprising grasses, sedges and broad-leaved weeds. The predominance of *E. crusgalli*, *C. dactylon*, *D. sanguinalis*, *C. rotundus*, *C. difformis* and several broad-leaved species reflects the complex weed spectrum commonly encountered in direct-seeded rice, where weeds emerge in close temporal proximity to the crop. Such simultaneous emergence increases the competitive pressure on rice during early establishment and makes reliance on a single herbicide mode of action less desirable. The progressive increase in weed density and biomass from 30 to 90 DAS under several herbicide treatments further suggests that early weed suppression alone may be insufficient to prevent subsequent weed recruitment and growth [11]. These observations emphasize the need for weed management programs that provide effective control across different weed groups and throughout critical stages of crop development [11]. This finding emphasizes the importance of sustained weed control rather than relying solely on early season suppression. The superior performance of T_4_ can be biologically linked to its sequential and complementary herbicide program. Pendimethalin applied pre-emergence provides early suppression of emerging weeds by interfering with cell division and elongation, thereby reducing initial weed establishment [12]. The subsequent application of florpyrauxifen-benzyl + penoxsulam extends control into the post-emergence period through two distinct physiological mechanisms: auxin-like disruption of susceptible broad-leaved weeds and ALS inhibition in susceptible weeds [13]. The combination therefore provides a broader weed control spectrum than treatments relying predominantly on a single biochemical target. This mechanism is consistent with the relatively lower density of grasses, sedges and particularly broad-leaved weeds under T_4_ throughout the observation period [14]. The comparatively weaker performance of T_2_ further highlights the importance of species-specific herbicide susceptibility. Although pendimethalin provided early suppression, the subsequent application of penoxsulam did not achieve weed control comparable to T_4_ under the prevailing field conditions. The greater densities of *C. rotundus* and *C. difformis* under T_2_ suggest that differences in weed susceptibility, emergence patterns, and growth stages may have contributed to treatment responses [15,16,17]. This finding indicates that herbicide efficacy in mixed weed communities depends not only on the active ingredients used but also on their spectrum of activity and the timing of application. Nevertheless, the response of these sedge species should be interpreted in the context of the particular weed populations and experimental conditions rather than as evidence of universal insensitivity to penoxsulam. The superior weed suppression observed with T_4_ therefore appears to be attributable to a better match between the herbicide spectrum of activity and the prevailing weed flora, rather than simply to a higher herbicide dose.

### 3.2. Weed Dry Weight

The pattern of weed dry matter provides stronger evidence of treatment efficacy than weed density alone, as biomass integrates both weed survival and subsequent growth. The progressive increase in weed dry weight from 30 to 90 DAS under the chemical treatments indicates that the surviving weeds continued to grow and exploit available resources as the crop developed. In contrast, the consistently lower weed biomass under T_4_ suggests that this treatment not only reduced weed establishment but also restricted the growth of weeds that escaped the initial control. The particularly low broad-leaved weed biomass under T_4_ is consistent with the inclusion of florpyrauxifen-benzyl, which disrupts normal auxin-regulated growth processes in susceptible weeds. Simultaneously, penoxsulam contributes to ALS-mediated inhibition of susceptible weed growth [15]. Such complementary physiological effects can reduce the probability that surviving weeds develop sufficient biomass to exert strong competition with rice [7,18]. The key implication is that effective weed management depends not only on reducing weed numbers but also on limiting biomass accumulation during crop development. This provides a plausible biological link between the lower weed biomass under T_4_ and its higher grain yield. Nevertheless, the relative contributions of reduced weed density and suppressed growth of surviving weeds cannot be separated without species-specific growth or weed–crop competition measurements.

### 3.3. Weed Control Efficiency and Weed Index

The WCE results integrated the effects observed in weed density and biomass and clearly demonstrated the superiority of T_4_. WCE under T_4_ remained high from 30 to 90 DAS, with only a modest reduction at the final observation. This sustained performance is important because the effectiveness of a weed management program should be evaluated not only by early suppression but also by its ability to prevent late-season weed resurgence. The superior performance of T_4_ is also supported by previous studies evaluating comparable herbicide combinations in rice. In rice, the post-emergence application of florpyrauxifen-benzyl + cyhalofop-butyl significantly reduced weed biomass and improved weed control efficiency and grain yield, with performance comparable to hand-weeding twice [19]. The relatively greater decline in WCE under T_2_ suggests inadequate residual and/or post-emergence control of the weed population during the later stages of crop growth. The relationship between WCE and weed index was particularly informative, as reduced weed suppression was associated with greater yield loss. T_4_ recorded the highest WCE and simultaneously, the lowest weed index among the chemical treatments. This indicates that greater suppression of weed population and biomass translated into greater protection of crop yield [20]. The biological basis is straightforward: reduced weed competition allows rice plants greater access to radiation, water, and nutrients, thereby sustaining photosynthetic activity and assimilate production during the stages critical for yield formation [21]. The very low weed index of T_4_ also indicates that its weed control performance approached the biological requirement necessary to protect rice yield from substantial weed-induced losses.

### 3.4. Yield

The yield response provides strong evidence that differences in weed suppression translated into agronomically meaningful outcomes. T_4_ produced 4.67 Mg/ha of grain, representing an 80.3% increase over the unweeded control and only a 2.7% reduction relative to the weed-free treatment (T6; 4.80 Mg/ha). This performance is consistent with findings from Indian direct-seeded rice systems, where florpyrauxifen-benzyl-based herbicide combinations improved weed control and grain yield [22,23]. The superior performance of T_4_ may be attributed to reduced competition for light, nutrients, water, and growing space, enabling rice plants to maintain canopy development and support assimilate production and grain filling. Similar benefits of combining ALS-inhibiting and synthetic auxin herbicides against mixed weed flora have been reported in transplanted rice by Venkatesh et al. [19]. The higher straw yield under T_4_ further supports improved vegetative growth, while the relatively narrow variation in harvest index suggests that weed management primarily influenced overall biomass production rather than substantially altering biomass partitioning. The 27.9% higher grain yield under T_4_ than under T_2_ further demonstrates the agronomic importance of selecting herbicide programs that effectively target the prevailing weed community. This advantage corresponded with lower weed density and biomass and higher weed control efficiency (WCE), suggesting that sustained suppression of competing weeds contributed to improved crop growth and productivity [22]. Nevertheless, the response should not be attributed exclusively to a single herbicide or mechanism, because the experiment evaluated complete treatment programs rather than the independent contributions of individual active ingredients. Regression analysis provided additional evidence of the relationship between weed pressure and crop performance: weed biomass at 90 DAS was negatively associated with grain yield and net returns, with *R*^2^ values of 0.909 and 0.853, respectively (Figure 5). These strong relationships indicate that treatments with lower weed biomass generally achieved greater productivity and profitability, although the associations alone do not establish causation. T_4_ occupied a favorable position by combining effective weed suppression with high grain yield and economic returns, highlighting the potential of sequential herbicide application to improve both agronomic performance and farm-level profitability in dry direct-seeded rice. However, its wider recommendation should consider herbicide and application costs, crop safety, environmental effects, and validation across locations and weed communities.

### 3.5. Economic Performance

The economic results demonstrate that biological effectiveness does not necessarily translate directly into economic superiority unless the additional cost of weed management is justified by the resulting yield gain. T_4_ achieved the highest net return and B:C ratio among the treatments because its superior weed control generated sufficient additional grain and straw yield to offset the cost of herbicide application. An important insight was obtained from comparison with the weed-free treatment. Although T_6_ produced slightly greater grain and gross returns, repeated hand weeding substantially increased cultivation costs. Consequently, T_4_ generated higher net returns and a markedly greater B:C ratio than complete manual weed control. This demonstrates a key principle of integrated weed management: the objective is not necessarily absolute weed eradication, but maintaining weed interference below the economic threshold while minimizing the cost of control [24]. The economic superiority of T_4_ therefore resulted from the convergence of three processes: broad-spectrum weed suppression, protection of crop yield and avoidance of high labor expenditure. This is particularly relevant to direct-seeded rice, where labor availability and cost can constrain repeated manual weeding. The results indicate that a well-timed sequential herbicide program can provide near-weed-free crop productivity without incurring the full cost of maintaining a completely weed-free field.

Taken together, the results indicate a consistent agronomic and economic response among the weed management treatments. T_4_ recorded lower weed infestation and weed biomass, high weed control efficiency, low yield loss, and grain yield close to that of the weed-free treatment, while also producing the highest net return and benefit–cost ratio. The consistency of these measured responses suggests that the performance of T_4_ was associated with effective suppression of the weed community throughout the crop growth period. However, the present study did not directly measure processes such as resource capture, resource-use competition, or crop–weed resource partitioning; therefore, the observed yield and economic responses are interpreted primarily in relation to the measured weed management indicators. The results further indicate that effective weed management in dry direct-seeded rice depends on maintaining adequate weed suppression across the crop season and addressing different weed groups, rather than simply increasing herbicide intensity. The integrated performance analysis provided a comparative visualization of the standardized agronomic and economic indicators, with T_4_ showing consistently high scores across the evaluated parameters (Figure 6). T_4_ showed a favorable combination of weed control efficiency, low weed index, grain yield, dry matter accumulation, net return, and benefit–cost ratio, indicating a balanced performance across the evaluated parameters. Thus, the advantage of T_4_ was reflected not only in weed suppression but also in the associated crop productivity and economic outcomes.

It is important to note that although the herbicide combination T_4_ showed promising efficacy for weed management and crop productivity, its sustainability should be interpreted with caution. The present study did not assess herbicide residues and persistence in soil, carry-over effects on succeeding crops, or potential environmental and ecological impacts. These aspects are important for evaluating the long-term safety and sustainability of herbicide-based weed management practices. Therefore, further research is warranted to assess soil residue dynamics, crop safety, environmental fate, and possible effects on non-target organisms and soil biological properties. Additionally, the study was conducted at a single location using one cultivar, which may limit the broader applicability of the findings. Treatment responses may vary with soil type, climatic conditions, agro-ecological conditions, and cultivar characteristics. Hence, multi-location and multi-season studies involving diverse cultivars and contrasting environments are needed to validate the consistency, environmental safety, and wider applicability of the observed responses and to support the long-term sustainability and extension of T_4_.

## 4. Materials and Methods

### 4.1. Experimental Site

The field experiment was conducted for two years during the rainy seasons of 2023 and 2024 at the experimental farm of Dr. Rajendra Prasad Central Agril. Univ., Pusa, in Samastipur, Bihar. The farm is located at 25°59′ N, 84°40′ E, at 52.9 m above mean sea level. The experimental site had a sub-tropical, sub-humid climate in the south-west monsoon region. During the study period, an average of 106 cm of rainfall was recorded, of which 80–90% occurred during June to October. The maximum and minimum temperatures were recorded at 30.0–35.6 °C and 21.0–26.0 °C, respectively, with mean values of 32.83 °C and 24.60 °C, respectively. Relative humidity ranged from 62 to 97%, while mean evaporation was 3.64 mm/day. The experimental soil was sandy loam in texture, containing 57.3% fine sand, 30.2% silt, and 12.5% clay, with a bulk density of 1.41 Mg/m^3^. The soil was alkaline in reaction (pH 8.38) with low organic carbon (0.41%) and low electrical conductivity (0.25 dS/m). The initial available nutrient status was 202 kg N/ha, 12.4 kg P/ha, and 110 kg K/ha.

### 4.2. Treatment Details

The experiment comprised seven weed management treatments. T_1_ consisted of pendimethalin 30% EC at 1000 g a.i./ha as pre-emergence, followed by bispyribac-sodium 10% SC at 25 g a.i./ha + pyrazosulfuron-ethyl 10% WP at 15 g a.i./ha at 25 DAS. T_2_ involved pendimethalin 30% EC at 1000 g a.i./ha as pre-emergence, followed by penoxsulam 21.7% SC at 20 g a.i./ha at 25 DAS. T_3_ consisted of pendimethalin 30% EC at 1000 g a.i./ha as pre-emergence, followed by triafamone 20% WG + ethoxysulfuron 10% WG at 63 g a.i./ha at 25 DAS. T_4_ comprised pendimethalin 30% EC at 1000 g a.i./ha as pre-emergence, followed by florpyrauxifen-benzyl 1.31% *w*/*w* at 15.3 g a.i./ha + penoxsulam 2.1% *w*/*w* at 20 g a.i./ha at 25 DAS. T_5_ involved pendimethalin 30% EC at 1000 g a.i./ha as pre-emergence, followed by penoxsulam 21.7% SC at 20 g a.i./ha at 12 DAS and bispyribac-sodium 10% SC at 25 g a.i./ha at 25 DAS. T_6_ was maintained weed-free through three-hand weeding, whereas T_7_ served as the unweeded control without any weed control treatment. The herbicides used in the experiment were sourced from the following manufacturers: pendimethalin from PI Industries Ltd. (Gurugram, India), bispyribac-sodium from Gharda Chemicals Ltd. (Mumbai, India), pyrazosulfuron-ethyl from United Phosphorus Ltd. (Mumbai, India), florpyrauxifen-benzyl and penoxsulam from Corteva Agriscience (Indianapolis, USA), and triafamone and ethoxysulfuron from Bayer Crop Science (Leverkusen, Germany). The current experiment was carried out in a randomized complete block design with 3 replications. A row-to-row distance of 20 cm was maintained. The gross and net plot sizes were 5 m × 4 m and 4.2 m × 3 m, respectively. The Rajendra Nilam variety of rice was sown for the study, which is a popular variety for DDSR cultivation since it was developed for aerobic conditions. Herbicides were applied at recommended doses using a knapsack sprayer with a flat fan nozzle at 500 L water/ha. The application of herbicides was done by maintaining adequate moisture in the field, and spraying was done under favorable weather conditions to avoid drift.

### 4.3. Weed Observations

Weed density, weed dry weight, weed control efficiency, and weed index were recorded to assess the effectiveness of different weed management treatments. Weed density was recorded species-wise at 30, 60, and 90 DAS using a 0.50 m^2^ quadrat placed randomly at 5 locations in each plot and expressed as number of weeds per m^2^, with weeds grouped into grasses, sedges, and broad-leaved weeds. For weed dry weight, weeds from the same quadrat were collected, washed, sun-dried, and oven-dried at 70 °C for 48 h, and the dry weight was expressed as g/m^2^. The weed density and dry weight data were subjected to √(x + 0.5) transformation before statistical analysis. The square-root transformation √(x + 0.5) was used to stabilize the variance and make weed density and dry weight data more normally distributed, thereby improving the validity and reliability of statistical analysis. WCE was calculated using the formula WCE (%) = [(WPC − WPT)/WPC] × 100, following Mani et al. [25], where WPC and WPT represent the weed population in the weedy control and treated plot, respectively. Weed index was calculated as Weed Index (%) = [(X − Y)/X] × 100, following Gill [26], where X represents grain yield in the weed-free plot, and Y represents grain yield in the treated plot.

### 4.4. Growth, Yield and Economics

Growth parameters, viz. plant height (cm), dry matter accumulation (g/m^2^), and tiller number (no./m^2^) were recorded at two critical growth stages of rice, viz. panicle initiation (PI) at 60 DAS and flowering to maturity at 90 DAS, for assessing the crop growth under different weed management treatments. Yield-attributing characters such as panicle number, grains per panicle, and test weight (G) were recorded before harvesting, whereas yield parameters were recorded after harvesting, sun-drying, and attainment of desired grain moisture content, i.e., 12–14%. Economics of the different treatments were worked out in terms of cost of cultivation (USD/ha), gross returns (USD/ha), net returns (USD/ha), and benefit: cost (B:C) ratio to find out the most suitable weed management treatment for higher productivity in the present study. The cost of cultivation for each treatment has been considered, which consists of the cost of all inputs and operations used in the experiment, including interest on working capital. The treatment-wise cost of weed management was USD 65.5, 52.9, 53.8, 75.5, 73.7, 206.3, and 0 per ha for T_1_, T_2_, T_3_, T_4_, T_5_, T_6_, and T_7_, respectively. The gross return was calculated from the price of grains (USD 23.6/Mg) and straw (USD 8.65/Mg) obtained from the respective treatments. Net returns were achieved by deducting the cost of cultivation from the gross returns, whereas the benefit: cost (B:C) ratio has been calculated as the ratio between net returns and cost of cultivation. The B:C ratio indicates the actual benefit generated for every unit of currency invested in each treatment.

### 4.5. Integrated Performance Analysis

An integrated performance analysis was performed to compare the overall agronomic and economic performance of the weed management treatments. Six indicators, viz. weed control efficiency at 90 DAS, inverse weed index, grain yield, net return, benefit: cost ratio, and dry matter accumulation at 90 DAS, were considered. Because these indicators differed in units and magnitude, the values were standardized to a 0–100 scale. For indicators in which higher values represented better performance (weed control efficiency, grain yield, net return, benefit: cost ratio, and dry matter accumulation), the standardized score was calculated as follows:

*Si* = (*Xi*/*X_max_*) ∗ *100*

where *Si* is the standardized score of a treatment, and *Xi* is its observed value, while *X_max_* is the maximum value among the treatments. For the weed index, where lower values indicate better weed management performance, the indicator was first converted to an inverse form and subsequently standardized so that a higher score represented better performance. Equal weight (1/6) was assigned to each of the six indicators, and the standardized scores were presented in a radar chart (Figure 6) to facilitate visual comparison of the relative performance of the treatments. The analysis was used for comparative visualization and was not intended to represent a separately measured biological variable.

### 4.6. Statistical Analysis

The data obtained from the experiment were subjected to analysis of variance (ANOVA) appropriate for a randomized complete block design (RCBD) following the procedure described by Snedecor and Cochran [27]. The significance of treatment effects was tested using the F-test at the 5% level of probability. When the treatment effect was significant, the least significant difference (LSD) at 5% probability was used for comparison among treatment means. The standard error of the mean (SEm±) was also calculated for each parameter. Before pooling the data from the two experimental years, Bartlett’s chi-square test was performed to assess the homogeneity of error variances between the years. Since the error variances were found to be homogeneous, pooled analysis was subsequently conducted following the procedure described by Cochran and Cox [28]. The ANOVA is included in the Supplementary Materials. Appropriate graphical representations made using MS Excel were used to present and facilitate interpretation of the experimental results.

## 5. Conclusions

The study revealed that effective and season-long weed suppression is critical for higher productivity and profitability of dry direct-seeded rice. In terms of herbicidal treatments, T_4_ (pendimethalin 1000 g/ha as pre-emergence followed by florpyrauxifen-benzyl + penoxsulam at 25 DAS) proved most effective in providing broad-spectrum weed control of grasses, sedges, and broad-leaved weeds. There was the lowest weed density and dry matter and the highest weed control efficiency (85.0–88.6%) and lowest weed index (2.7%) in T_4_. As a result, the treatment reported the highest grain yield of 4.67 Mg/ha, which was statistically comparable with the weed-free control (4.80 Mg/ha) but 27.9% higher than that of T_2_. It also recorded the highest net return (USD 657/ha) and B:C ratio (1.62), implying that the superior biological efficiency of the treatment translated into economic gains. The results suggest that sequential application of herbicides with complementary modes of action can provide effective weed management and maintain rice productivity while reducing reliance on labor-intensive manual weeding. Under the specific experimental conditions evaluated, this herbicide sequence represents a potential weed management option for dry direct-seeded rice. Further validation across locations, seasons, and cultivars is required before extending this recommendation to other production environments.

## Figures and Tables

**Figure 1:**
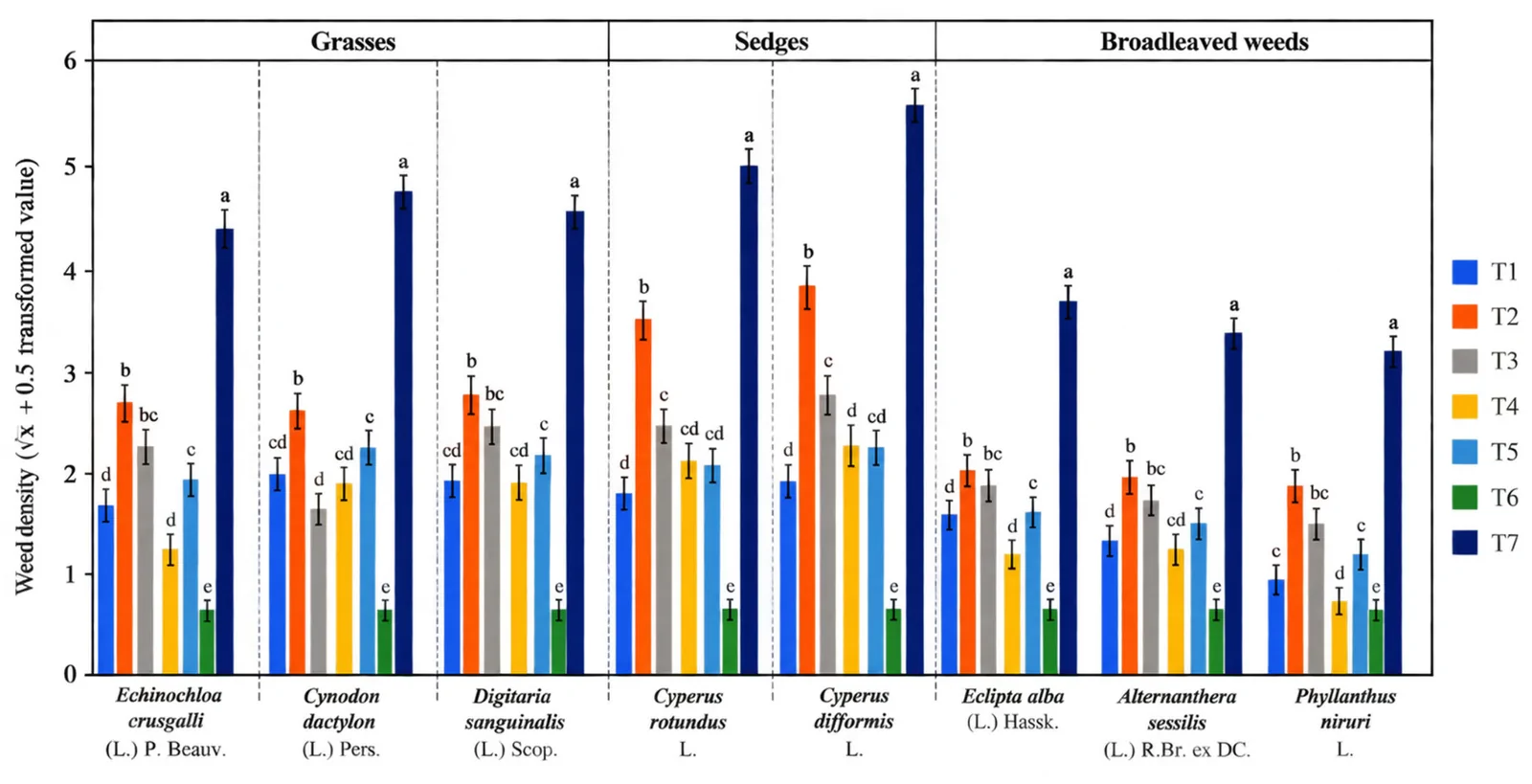
Weed density (number/m^2^) at 30 DAS under different herbicidal applications in DDSR cultivation (pooled over 2 years). [T_1_—pendimethalin at 1000 g/ha as PRE *fb* bispyribac sodium at 25 g/ha + pyrazosulfuron ethyl at 15 g/ha at 25 DAS as POST; T_2_—pendimethalin at 1000 g/ha as PRE *fb* penoxsulam at 20 g/ha as POST at 25 DAS; T_3_—pendimethalin at 1000 g/ha as PRE *fb* triafamone 20% WG + ethoxysulfuron 10% WG at 63 g/ha at 25 DAS as POST; T_4_—pendimethalin at 1000 g/ha as PRE *fb* florpyrauxifen-benzyl 1.31% *w*/*w* at 15.3 g/ha + penoxsulam 2.1% *w*/*w* at 20 g/ha at 25 DAS as POST; T_5_—pendimethalin at 1000 g/ha as PRE *fb* penoxsulam at 20 g/ha as early POST at 12 DAS *fb* bispyribac sodium at 25 g/ha as POST at 25 DAS; T_6_—weed-free (three-hand weeding); and T_7_—unweeded control; data in parentheses (original value) was subjected to √(*x* + 0.5) transformation; different letters above bars denote statistical significance, otherwise at par.]

**Figure 2:**
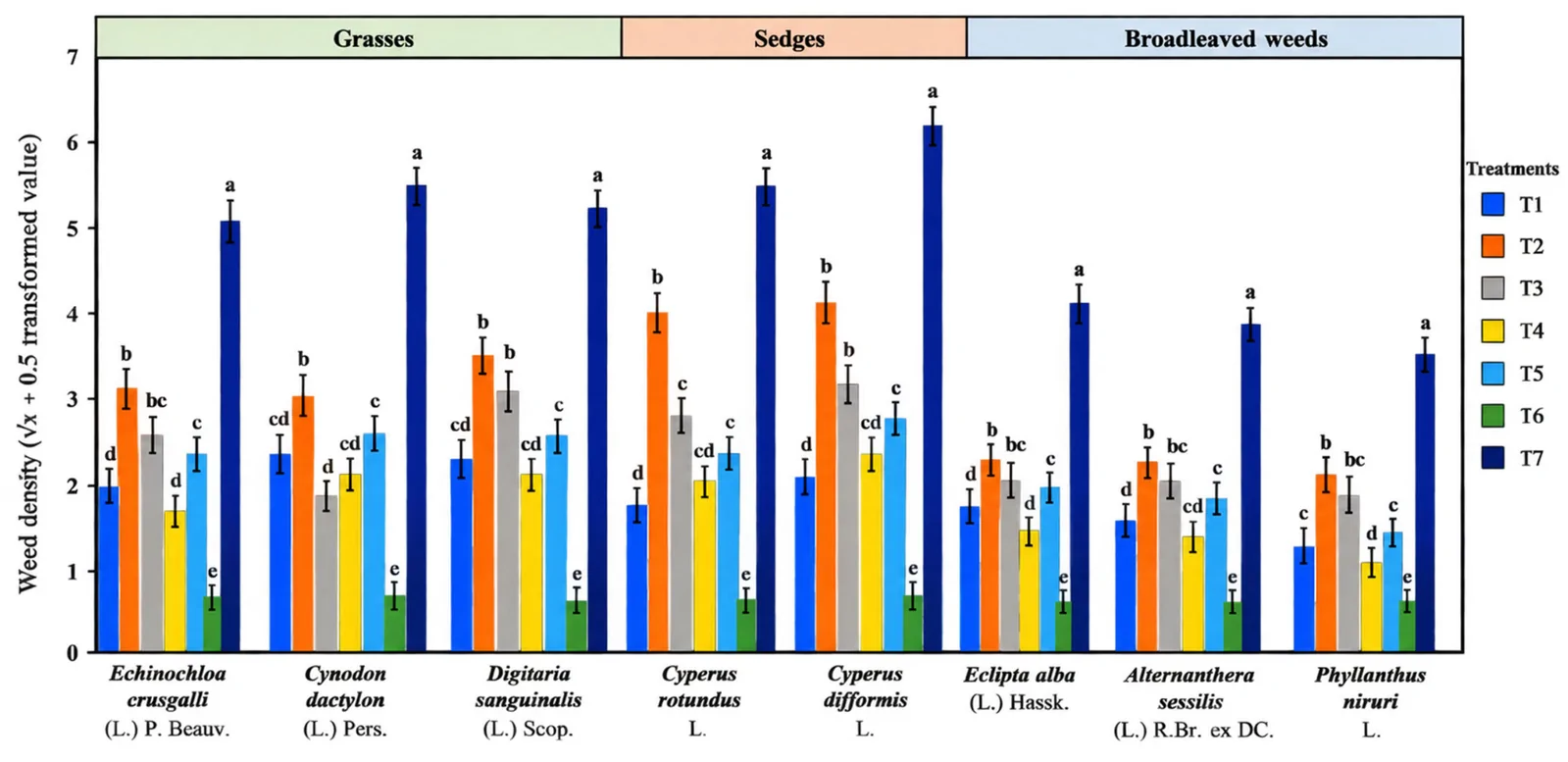
Weed density (number/m^2^) at 60 DAS under different herbicidal applications in DDSR cultivation (pooled over 2 years). [Treatment details are the same as mentioned below Figure 1; data in parentheses (original value) were subjected to √(*x* + 0.5) transformation; different letters above bars denote statistical significance, otherwise at par.]

**Figure 3:**
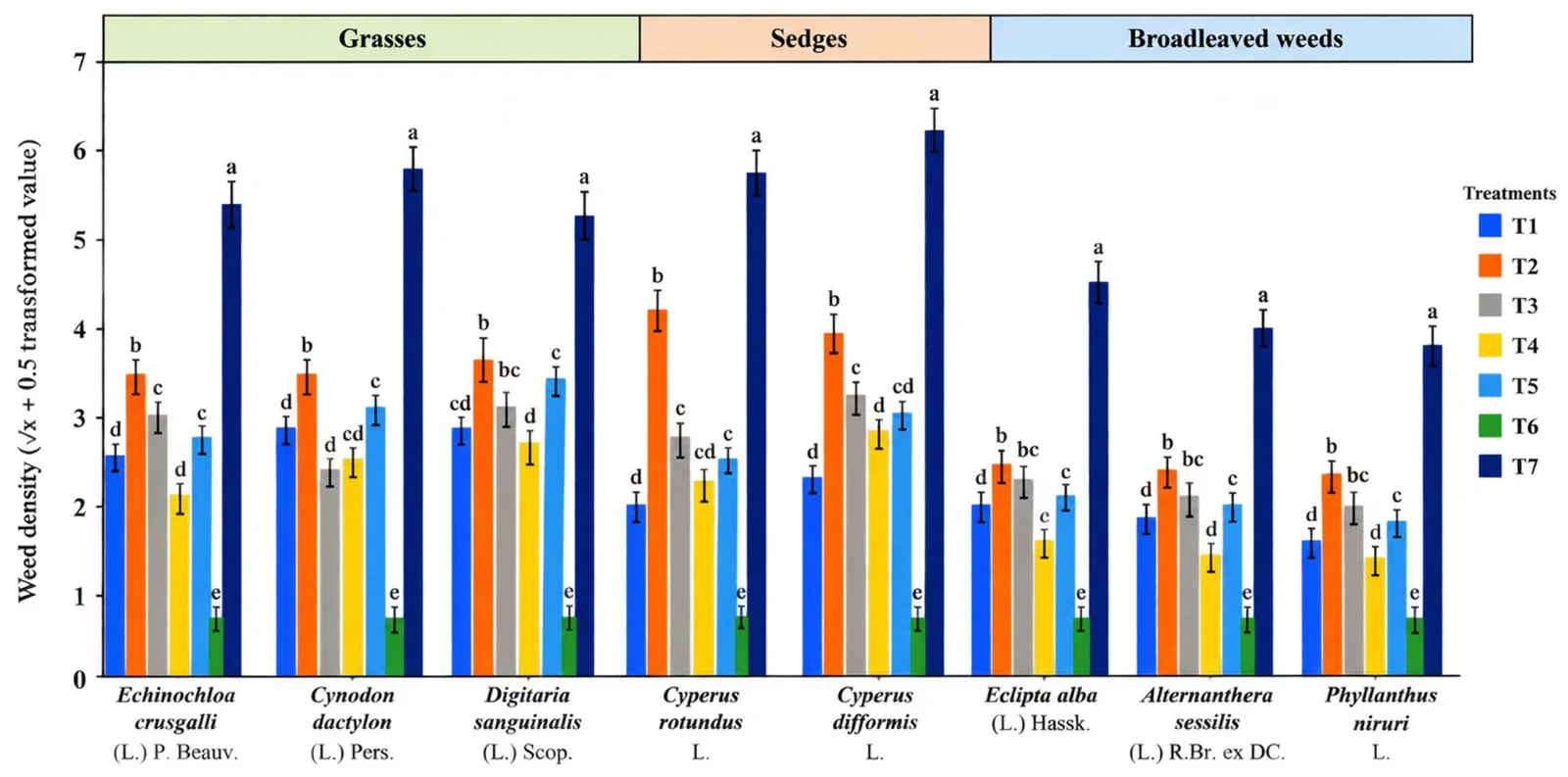
Weed density (number/m^2^) at 90 DAS under different herbicidal applications in DDSR cultivation (pooled over 2 years). [Treatment details are the same as mentioned below Figure 1; data in parentheses (original value) were subjected to √(*x* + 0.5) transformation; different letters above bars denote statistical significance, otherwise at par.]

**Figure 4:**
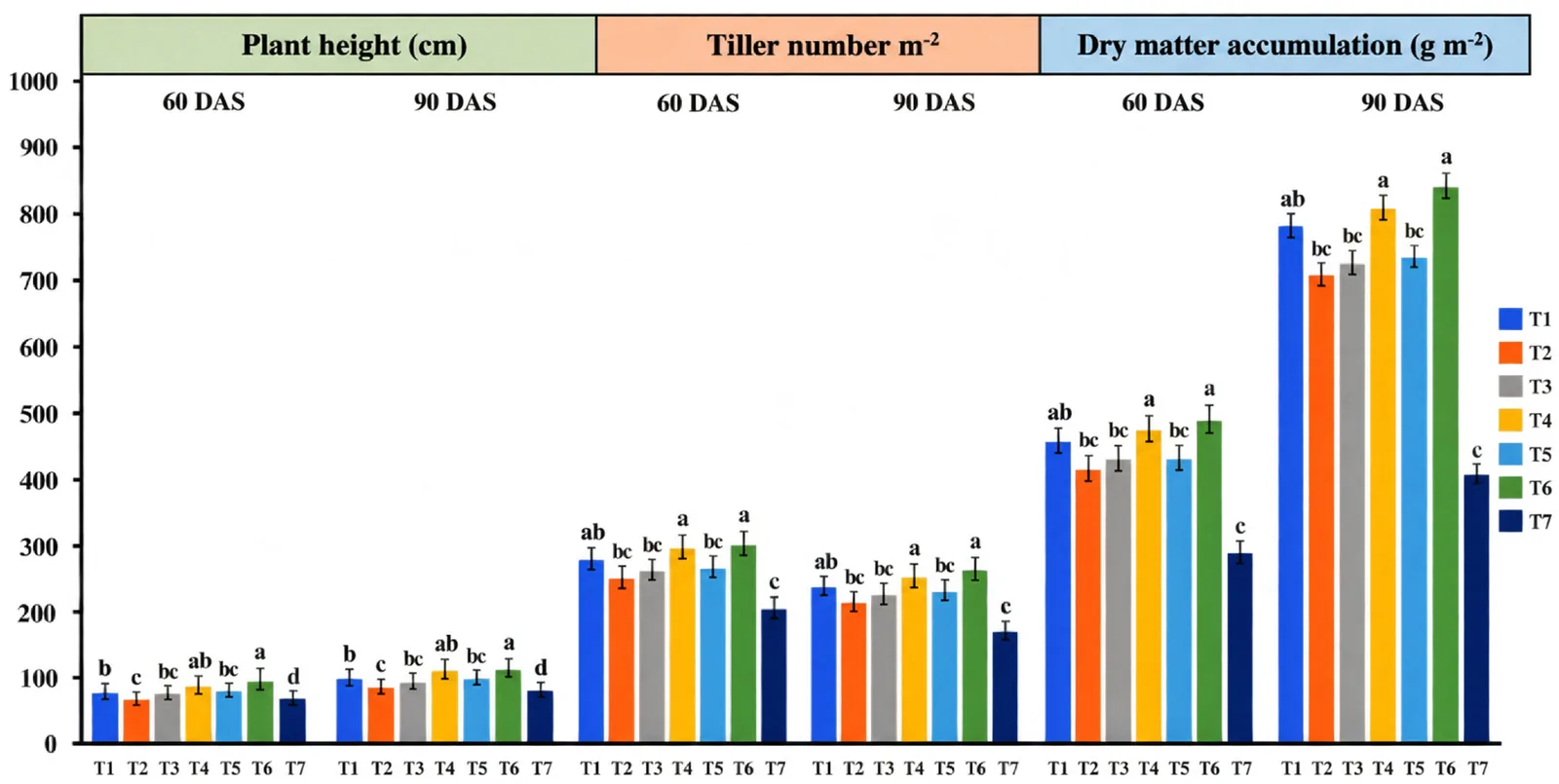
Growth of DDSR under different herbicide applications (pooled over 2 years). [Treatment details are the same as mentioned below Figure 1; different letters above bars denote statistical significance, otherwise at par.]

**Figure 5:**
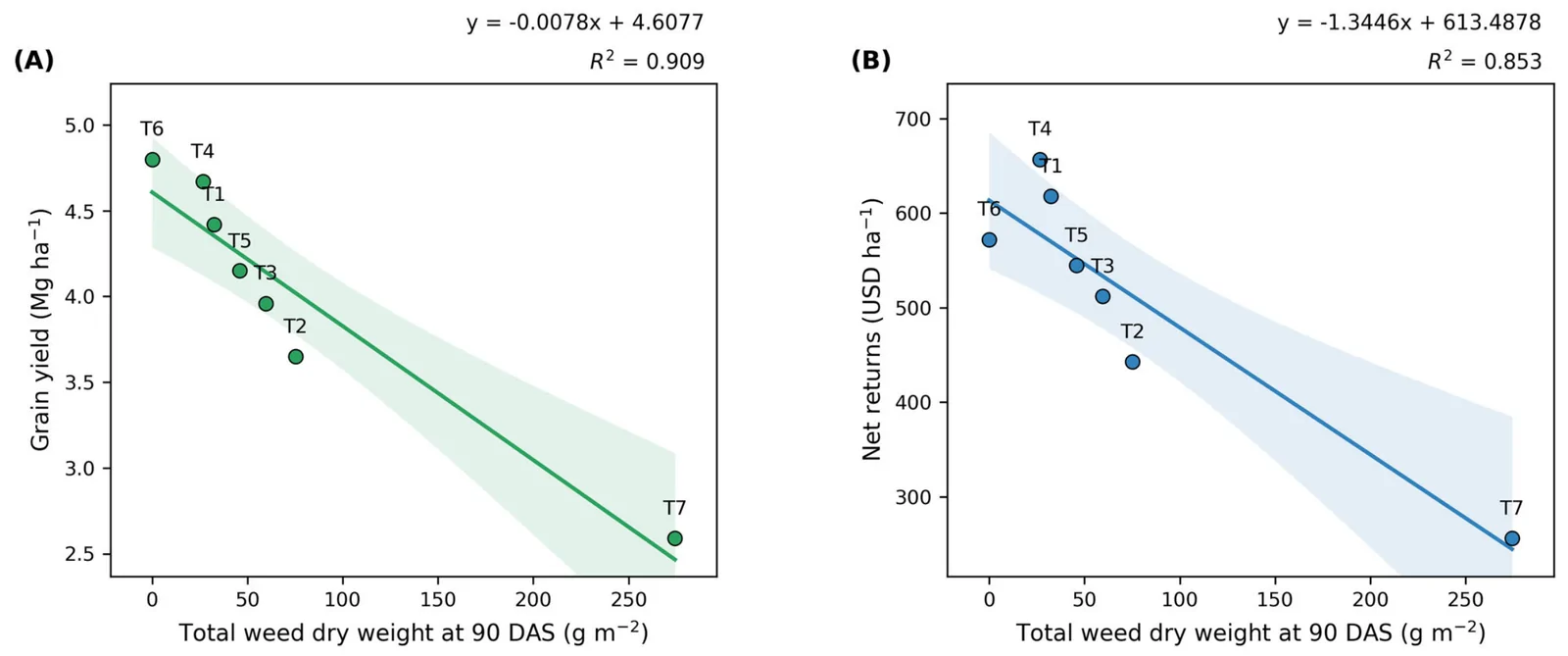
Relationship between weed dry weight at 90 DAS and (**A**) grain yield as well as (**B**) net returns.

**Figure 6:**
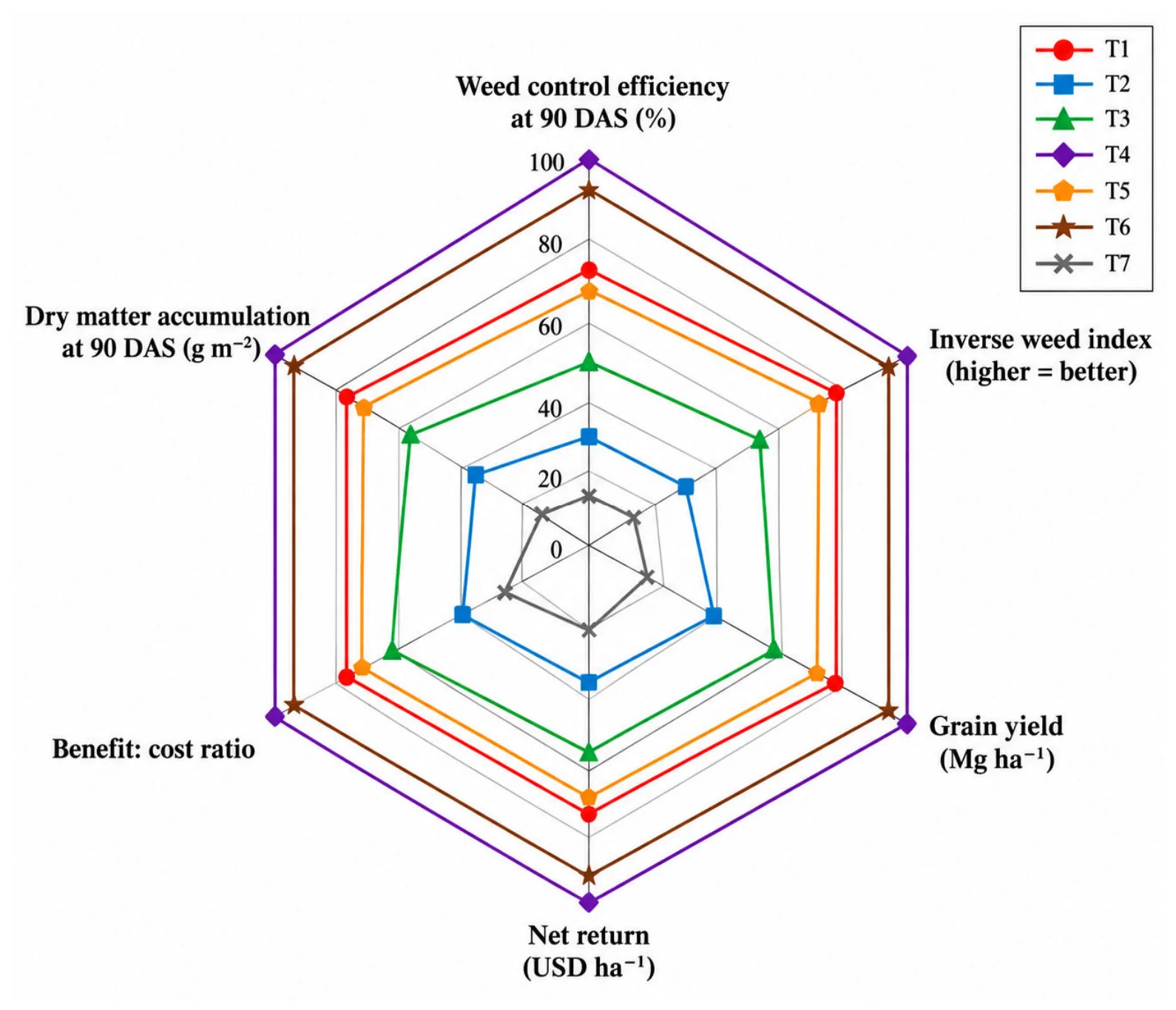
Integrated agronomic and economic performance of weed management strategies in dry direct-seeded rice.

**Table 1:** Effect of different herbicide applications on weed dry weight (g/m^2^) under DDSR cultivation at different growth stages (pooled over two years).

Treatments	Grasses			Sedges			Broad-Leaved Weeds		
	30 DAS	60 DAS	90 DAS	30 DAS	60 DAS	90 DAS	30 DAS	60 DAS	90 DAS
T_1_	2.90 cd (8)	3.76 bc (14)	3.99 bc (15.99)	2.15 d (4.28)	2.88 c (8.0)	3.13 d (9.77)	1.87 c (3.13)	2.42 cd (5.54)	2.62 cd (6.61)
T_2_	3.97 b (15.41)	4.83 b (23.16)	5.54 b (30.77)	4.01 b (15.81)	5.06 b (25.52)	5.40 b (29.07)	2.78 b (7.44)	3.59 b (12.77)	3.92 b (15.42)
T_3_	3.67 bc (13.11)	4.44 b (19.73)	5.12 b (26.17)	3.26 bc (10.28)	4.13 bc (16.99)	4.73 bc (22.11)	2.39 bc (5.43)	3.08 bc (9.17)	3.40 bc (11.34)
T_4_	2.30 d (5.02)	2.73 c (7.47)	3.15 c (9.91)	2.49 cd (5.91)	3.27 c (10.55)	3.63 c (12.94)	1.67 c (2.43)	1.94 d (3.32)	2.06 d (3.84)
T_5_	3.35 bc (10.86)	4.06 b (16.42)	4.64 bc (21.34)	2.81 cd (7.61)	3.54 c (12.56)	4.02 cd (16.24)	2.08 bc (3.99)	2.71 bcd (6.94)	2.92 bcd (8.36)
T_6_	0.71 e (0)	0.71 d (0)	0.71 d (0)	0.71 e (0)	0.71 d (0)	0.71 e (0)	0.71 d (0)	0.71 e (0)	0.71 e (0)
T_7_	6.62 a (43.74)	9.15 a (83.59)	10.46 a (109.79)	5.97 a (35.60)	8.84 a (78.29)	10.26 a (105.3)	5.35 a (20.30)	6.55 a (42.63)	7.71 a (59.23)
SEm (±)	0.31	0.44	0.54	0.34	0.47	0.45	0.30	0.31	0.38
LSD (*p* = 0.05)	0.93	1.31	1.59	1.00	1.38	1.34	0.89	0.91	1.13

[Treatment details are the same as mentioned below Figure 1; data in parentheses (original value) were subjected to √(*x* + 0.5) transformation; different letters denote statistical significance.]

**Table 2:** Effect of different herbicide applications on weed control efficiency and weed index (mean over two years).

Treatments	Weed Control Efficiency (%)			Weed Index (%)
	30 DAS	60 DAS	90 DAS	
T_1_	84.7 ± 5.5	85.6 ± 4.5	79.6 ± 5.2	7.9 ± 0.5
T_2_	68.3 ± 3.9	69.9 ± 6.8	59.0 ± 6.7	24.0 ± 0.1
T_3_	74.9 ± 6.7	78.0 ± 7.7	70.1 ± 1.2	17.5 ± 0.8
T_4_	88.0 ± 4.4	88.6 ± 2.5	85.0 ± 5.0	2.7 ± 0.1
T_5_	79.1 ± 3.9	81.8 ± 3.9	73.4 ± 2.8	13.5 ± 0.5
T_6_	100 ± 0.0	100 ± 0.0	100 ± 0.0	-
T_7_	-	-	-	46.0 ± 1.1

[Treatment details are the same as mentioned below Figure 1; mean ± SD (*n* = 6).]

**Table 3:** Yield attributes of DDSR under different herbicide applications (pooled over two years).

Treatments	Panicle Numbers/m^2^	Grains/Panicle	Test Weight (g)
T_1_	222 ab	131 ab	23.2 a
T_2_	197 b	121 b	22.5 a
T_3_	202 b	122 b	23.1 a
T_4_	236 a	136 ab	23.6 a
T_5_	208 b	126 b	23.0 a
T_6_	240 a	144 a	23.9 a
T_7_	151 c	86 c	22.2 a
SEm (±)	8	6	1
LSD (*p* = 0.05)	25	17	NS

[Treatment details are the same as mentioned below Figure 1; different letters denote statistical significance.]

**Table 4:** Yield parameters of DDSR under different herbicide applications (pooled over two years).

Treatments	Grain Yield (Mg/ha)	Grain Yield Increase Over Unweeded Control (%)	Straw Yield (Mg/ha)	Harvest Index (%)
T_1_	4.42 bc	70.7	5.92 ab	42.7 a
T_2_	3.65 e	40.9	5.03 e	42.1 a
T_3_	3.96 de	52.9	5.45 c	42.1 a
T_4_	4.67 ab	80.3	6.01 ab	43.7 a
T_5_	4.15 cd	60.2	5.65 bc	42.3 a
T_6_	4.80 a	85.3	6.22 a	43.6 a
T_7_	2.59 f	-	3.85 f	40.2 b
SEm (±)	0.12	-	0.14	0.6
LSD (*p* = 0.05)	0.35	-	0.40	1.7

[Treatment details are the same as mentioned below Figure 1; different letters denote statistical significance.]

**Table 5:** Economic analysis of different treatments (pooled over two years).

Treatments	Cost of Cultivation (USD/ha)	Gross Returns (USD/ha)	Net Returns (USD/ha)	B:C Ratio
T_1_	394	1011 bc	618 ab	1.57 ab
T_2_	384	827 e	443 d	1.15 cd
T_3_	393	905 de	512 cd	1.30 cd
T_4_	406	1063 b	657 a	1.62 a
T_5_	404	950 cd	545 bc	1.35 bc
T_6_	533	1105 a	572 abc	1.07 d
T_7_	332	588 f	256 e	0.77 e
	-	33	33	0.08
LSD (*p* = 0.05)	-	99	99	0.25

[Treatment details are the same as mentioned below Figure 1; different letters denote statistical significance.]

## Data Availability

Data will be available on request.

## Supplementary Materials

The following supporting information can be downloaded at https://www.mdpi.com/article/10.3390/plants15213486/s1, Table S1: ANOVA table for species-wise grassy weed count (no./m^2^); Table S2: ANOVA table for species-wise sedge and broadleaved weed count (no./m^2^); Table S3: ANOVA table for weed dry weight of grassy, sedge, and broadleaved weeds (g/m^2^); Table S4: ANOVA table for growth attributes of rice; Table S5: ANOVA table for yield and yield attributes of rice; Table S6: ANOVA table for economic analysis; Table S7: Detailed cost of cultivation (USD/ha).

## References

[1] Pramanick B., Kumar M., Naik B.M., Singh S.K., Kumar M., Singh S. (2024). Soil carbon-nutrient cycling, energetics, and carbon footprint in calcareous soils with adoption of long-term conservation tillage practices and cropping systems diversification. Sci. Total Environ..

[2] Saha S., Munda S., Singh S., Kumar V., Jangde H.K., Mahapatra A., Chauhan B.S. (2021). Crop establishment and weed control options for sustaining dry direct seeded rice production in eastern India. Agronomy.

[3] Shekhawat K., Rathore S.S., Chauhan B.S. (2020). Weed management in dry direct-seeded rice: A review on challenges and opportunities for sustainable rice production. Agronomy.

[4] Banik N.C., Kumar A., Mohapatra B.K., Kumar V., Sreenivas C., Singh S., Panneerselvam P., Kumar V. (2020). Weed management approaches in direct-seeded rice in eastern Indian ecologies—A critical and updated review. Indian J. Weed Sci..

[5] Mahajan G., Ramesha M.S., Chauhan B.S. (2014). Response of rice genotypes to weed competition in dry direct-seeded rice in India. Sci. World J..

[6] Rao A.N., Johnson D.E., Sivaprasad B., Ladha J.K., Mortimer A.M. (2007). Weed management in direct-seeded rice. Adv. Agron..

[7] Wu M., Ou M., Zhang Y., Jia W., Dai S., Wang M., Dong X., Wang X., Jiang L. (2024). Development and Evaluation of a Monodisperse Droplet-Generation System for Precision Herbicide Application. Agriculture.

[8] Singh R., Pramanick B., Singh A.P., Neelam, Kumar S., Kumar A., Singh G.D. (2017). Bio-efficacy of Fenoxaprop-P-Ethyl for grassy weed control in onion and its residual effect on succeeding maize crop. Indian J. Weed Sci..

[9] Kaur T., Kaur S., Singh M., Singh G., Gill J.S., Kaur P., Bhullar M.S. (2025). Effective weed management under direct seeded rice through use of florpyrauxifen-benzyl plus cyhalofop-butyl as post-emergence herbicide in northwestern India. Indian J. Agron..

[10] Singh R., Pramanick B., Kumar S., Pal S., Prabhakar D., Naik B.M., Naik B.S.S.S., Alasmari A., Znazen H., Gaber A. (2026). Different weed management practices influence weed dynamics and productivity of maize (*Zea mays* L.). Phytoparasitica.

[11] Shehzad A., Dilshad M., Shahzadi K., Ghani A., Ibrar I., Ahmed N., Yasin M., Ali B., Ali B., Nayab S.F. (2026). Evaluating the Efficacy of Different Chemical Treatments on Weed Density, Biomass Accumulation, and Physiological Traits in Pearl Millet. Int. J. Adv. Sustain. Dev..

[12] Pamungkas U.R.R., Chankaew S., Jongrungklang N., Monkham T., Gonkhamdee S. (2025). The efficacy of pre-emergence herbicides against dominant soybean weeds in Northeast Thailand. Agronomy.

[13] Inci D., Al-Khatib K. (2026). Seasonwide weed management utilizes florpyrauxifen-benzyl in water-seeded rice production systems. Agrochemicals.

[14] Zhang Z., Becerra-Alvarez A., Al-Khatib K. (2025). Physiological Action of Bioherbicides in Weed Control: A Systematic Review. Front. Agron..

[15] Singh V.P., Singh S.P., Tripathi N., Singh M.K., Kumar A. (2009). Bioefficacy of penoxsulam on transplanted rice weeds. Indian J. Weed Sci..

[16] Yadav D.B., Singh N., Kumar J., Yadav A. (2019). Penoxsulam + butachlor: A new ready-mix herbicide for control of complex weed flora in transplanted rice. Indian J. Weed Sci..

[17] Guo Y., Nan S., Song C., Liu Y., Ma H., Wu B. (2026). Enhanced Inactivation of Aspergillus flavus in Rice by Combined Physical Technologies with Tempering: Efficacy and Quality Evaluation. Food Bioprocess Technol..

[18] Hashim M., Singh V.K., Singh K.K., Dhar S. (2022). Herbicidal weed-management options for dry direct-seeded rice (*Oryza sativa*) in North-Eastern Plains of India. Indian J. Agron..

[19] Venkatesh B., Parameswari Y.S., Madhavi M., Ram Prakash T. (2021). Performance of herbicides and herbicide mixtures on weed control in transplanted rice. Indian J. Weed Sci..

[20] Upadhaya B., Tiwari R.K., Kishor K., Harsha B.R., Singh S.K., Kumar S., Kumar S. (2024). Herbicide evaluation in direct seeded rice in the middle Gangetic plains of India. Weed Biol. Manag..

[21] Jarin A.S., Islam M.M., Rahat A., Ahmed S., Ghosh P., Murata Y. (2024). Drought stress tolerance in rice: Physiological and biochemical insights. Int. J. Plant Biol..

[22] Arockia Infant Paul R., Murali Arthanari P., Peramaiyan P., Kumar V., Bagavathiannan M., Sabarivasan R. (2025). UAV-based herbicide application for efficient weed control and resource savings in direct-seeded rice. Crop Prot..

[23] Karthickraja A., Saravanane P., Poonguzhalan R., Nadaradjan S. (2024). Comparison of UAV and knapsack herbicide application methods on weed spectrum, crop growth and yield in dry direct-seeded rice. Indian J. Weed Sci..

[24] Ahmed S., Kumar V., Zaman A.U., Dewan M.R., Khatun A., Hossain K., Singh S., Timsina J., Krupnik T.J. (2025). Dry direct-seeded and broadcast rice: A profitable and climate-smart alternative to puddled transplanted *aus* rice in Bangladesh. Field Crops Res..

[25] Mani V.S., Mala M.L., Gautam K.C., Bhagavandas (1973). Weed killing chemicals in potato cultivation. Indian Farming.

[26] Gill G.S., Kumar V. (1969). Weed index—A new method of reporting weed control trials. Indian J. Agron..

[27] Snedecor G.W., Cochran W.G. (1967). Statistical Methods.

[28] Cochran W.G., Cox G.M. (1957). Experimental Designs.

