# Supplementary material for: Exploration of New-Generation Herbicide Combination for Direct-Seeded Rice Cultivation in India
Source: Plants (Basel). 2026 Nov 8;15(21):3486. doi: 10.3390/plants15213486 (PMC13766191; doi:10.3390/plants15213486)
Supplement: Supplementary file 1 [file plants-15-03486-s001.zip › plants-4585469-supplementary.pdf]

Supplementary Table S1. ANOVA table for species-wise grassy weed count (no./m²)

Source of variation	DF	F-calculated									F-tabulated	Significance
		E. crussgalli at 30 DAS	*C. dactylon* at 30 DAS	*D. sanguinalis* at 30 DAS	*E. crussgalli* at 60 DAS	*C. dactylon* at 60 DAS	*D. sanguinalis* at 60 DAS	*E. crussgalli* at 90 DAS	*C. dactylon* 90 DAS	*D. sanguinalis* 90 DAS		
Year (Y)	1	1.76	0.71	0.83	1.55	0.65	0.77	1.79	1.11	0.82	4.26	NS
Replication within Year	4	1.05	0.55	0.21	0.59	0.33	0.51	1.04	1.06	0.24	2.78	NS
Treatment (T)	6	58.03	38.98	30.86	50.45	35.21	28.25	59.86	45.54	25.55	2.51	S (*)
Year × Treatment (Y × T)	6	0.55	0.21	1.17	0.98	0.78	0.25	0.67	1.05	1.31	2.51	NS
Error	24	-	-	-	-	-	-	-	-	-	-	-

S(*) = significant; NS = non-significant

Supplementary Table S2. ANOVA table for species-wise sedge and broadleaved weed count (no./m²)

Source of variation	DF	F-calculated															F-tabulated	Significance
		C. rotundus at 30 DAS	*C. difformis* at 30 DAS	*E. alba* at 30 DAS	*A. sessilis* at 30 DAS	*P. niruni* at 30 DAS	*C. rotundus* at 60 DAS	*C. difformis* at 60 DAS	*E. alba* at 60 DAS	*A. sessilis* at 60 DAS	*P. niruni* at 60 DAS	*C. rotundus* at 90 DAS	*C. difformis* at 90 DAS	*E. alba* at 90 DAS	*A. sessilis* at 90 DAS	*P. niruni* at 90 DAS		
Year (Y)	1	1.25	1.11	1.75	1.02	0.98	1.55	1.67	0.78	1.55	1.21	1.19	1.26	1.88	1.50	1.33	4.26	NS
Replication within Year	4	0.75	2.15	1.08	0.76	0.55	1.17	1.19	0.59	1.08	1.11	1.01	0.89	1.33	1.05	0.78	2.78	NS
Treatment (T)	6	28.55	55.85	78.21	25.86	33.25	65.37	25.88	48.98	63.78	70.52	29.98	33.45	36.78	27.65	36.45	2.51	S (*)
Year × Treatment (Y × T)	6	0.78	0.35	0.98	0.55	0.21	1.06	1.15	0.24	0.78	0.65	0.28	0.65	0.75	0.21	0.33	2.51	NS
Error	24	-	-	-	-	-	-	-	-	-	-	-	-	-	-	-	-	-

S(*) = significant; NS = non-significant

Supplementary Table S3. ANOVA table for weed dry weight of grassy, sedge, and broadleaved weeds (g/m^2)

Source of variation	DF	F-calculated									F-tabulated	Significance
		Grass at 30 DAS	Sedge at 30 DAS	Broadleaf at 30 DAS	Grass at 60 DAS	Sedge at 60 DAS	Broadleaf at 60 DAS	Grass at 90 DAS	Sedge at 90 DAS	Broadleaf at 90 DAS		
Year (Y)	1	1.15	0.98	0.85	0.67	1.15	2.05	1.95	1.88	1.77	4.26	NS
Replication within Year	4	0.39	0.40	0.27	0.91	0.39	0.91	0.23	0.74	0.90	2.78	NS
Treatment (T)	6	35.31	25.68	16.46	36.57	31.23	37.73	33.10	45.38	35.74	2.51	S (*)
Year × Treatment (Y × T)	6	1.52	1.24	1.05	0.67	0.78	0.92	0.99	1.01	1.11	2.51	NS
Error	24	-	-	-	-	-	-	-	-	-	-	-

S(*) = significant; NS = non-significant

Supplementary Table S4. ANOVA table for growth attributes of rice

Source of variation	DF	F-calculated						F-tabulated	Significance
		Plant height (cm) at 60 DAS	Plant height (cm) at 90 DAS	Tiller number/m^2 at 60 DAS	Tiller number/m^2 at 90 DAS	Dry matter accumulation (g/m^2) at 60 DAS	Dry matter accumulation (g/m^2) at 90 DAS		
Year (Y)	1	3.33	2.25	1.56	1.99	2.31	3.51	4.26	NS
Replication within Year	4	0.03	0.24	4.56*	4.99*	0.34	0.96	2.78	NS
Treatment (T)	6	7.69	10.94	233.56	255.86	19.31	27.68	2.51	S (*)
Year × Treatment (Y × T)	6	1.33	1.07	1.89	0.98	0.55	0.68	2.51	NS
Error	24	-	-	-	-	-	-	-	-

S(*) = significant; NS = non-significant; *Significant

Supplementary Table S5. ANOVA table for yield and yield attributes of rice

Source of variation	DF	F-calculated						F-tabulated	Significance
		Panicle per m^2	Grains per panicle	Test weight (g)	Grain yield (Mg/ha)	Straw yield (Mg/ha)	HI (%)		
Year (Y)	1	1.25	0.89	0.55	0.67	0.33	1.15	4.26	NS
Replication within Year	4	0.32	0.97	0.38	0.37	0.52	0.82	2.78	NS
Treatment (T)	6	11.89	11.55	0.72**	24.93	24.26	3.82	2.51	S (*)
Year × Treatment (Y × T)	6	1.05	1.99	0.57	0.96	0.78	0.71	2.51	NS
Error	24	-	-	-	-	-	-	-	-

S(*) = significant; NS = non-significant; **Non-significant

Supplementary Table S6. ANOVA table for economic analysis

Source of variation	DF	F-calculated			F-tabulated	Significance
		Gross returns	Net returns	B:C ratio		
Year (Y)	1	1.77	1.77	1.62	4.26	NS
Replication within Year	4	0.41	0.41	0.31	2.78	NS
Treatment (T)	6	28.10	28.10	12.13	2.51	S (*)
Year × Treatment (Y × T)	6	1.09	1.09	0.85	2.51	NS
Error	24	-	-	-	-	-

S(*) = significant; NS = non-significant

Supplementary Table S7. Detailed cost of cultivation (USD/ha)

Treatments	Labour cost	Herbicide cost
T1	163	65.5
T2	163	52.9
T3	163	53.8
T4	163	75.5
T5	163	73.7
T6	369.3	0
T7	163	0
